# Protective effects of methanolic leaf extracts of *Monanthotaxis caffra* against aflatoxin B1-induced hepatotoxicity in rats

**DOI:** 10.4102/ojvr.v89i1.1968

**Published:** 2022-03-23

**Authors:** Rhulani Makhuvele, Kenn Foubert, Nina Hermans, Luc Pieters, Luc Verschaeve, Esam Elgorashi

**Affiliations:** 1 Toxicology and Ethnoveterinary Medicine, Agricultural Research Council-Onderstepoort Veterinary research, Onderstepoort, South Africa; 2 Department of Paraclinical Sciences, Faculty of Veterinary Science, University of Pretoria, Onderstepoort, South Africa; 3 Department of Pharmaceutical Sciences, University of Antwerp, Antwerp, Belgium; 4 Department of Risk and Health Impact Assessment, Sciensano, Brussels, Belgium; 5 Department of Biomedical Sciences, University of Antwerp, Antwerp, Belgium

**Keywords:** mycotoxins, aflatoxins, Annonaceae, liver toxicity, liver enzymes, amelioration

## Abstract

Aflatoxins are potent hepatotoxic and carcinogenic secondary metabolites produced by toxigenic fungi. The present study investigated the protective effect of methanolic leaf extracts of *Monanthotaxis caffra* (MLEMC) against aflatoxin B1-induced toxicity in male Sprague-Dawley rats. The rats were randomly divided into 6 groups of 8 animals each. Five groups were administered orally for seven days with three different concentrations of MLEMC (100 mg/kg, 200 mg/kg and 300 mg/kg), curcumin (10 mg/kg) or vehicle (25% propylene glycol). The following day, these groups were administered 1 mg/kg b.w. of aflatoxin B_1_ (AFB_1_). The experiment was terminated three days after administration of AFB_1_. Group 6 represented untreated healthy control. Serum aspartate aminotransferase, alanine aminotransferase, alkaline phosphatase, lactate dehydrogenase, creatinine and liver histopathology were evaluated. Methanolic leaf extracts of *M. caffra* decreased the levels of aspartate aminotransferase, alanine aminotransferase, lactate dehydrogenase and creatinine in the sera of rats as compared with the AFB_1_ intoxicated group. Co-administration of MLEMC improved the histological characteristics of the hepatocytes in contrast to the AFB_1_ treated group, which had mild to severe hepatocellular injuries including bile duct proliferation, bile duct hyperplasia, lymphoplasmacytic infiltrate and fibrosis. Extracts of *M. caffra* were beneficial in mitigating the hepatotoxic effects of AFB_1_ in rats by reducing the levels of liver enzymes and preventing hepatic injury.

## Introduction

Aflatoxins (AFs) are group of difuranocoumarins toxic secondary metabolites produced by *Aspergillus flavus, Aspergillus parasiticus* and *Aspergillus nomius,* which contaminate various foodstuff and feed. The most predominant major AFs that contaminate food and feed commodities include AFB_1_, AFB_2_, AFG_1_ and AFG_2_ and their hydroxylated metabolites, namely AFM_1_ (Ráduly et al. 2020). Aflatoxin B_1_ is the most potent hepatotoxin, mutagen, teratogen, immunosuppressive and carcinogen amongst other major groups of AFs in nature (Zarev et al. 2020). When ingested, it is bio-transformed in the liver primarily by CYP3A4 and CYP1A2 isoforms that are members of the cytochrome P450 (CYP) superfamily of drug metabolising enzymes and generate a highly reactive species, namely AFB_1_-exo-epoxide and other metabolites (Bedard & Massey 2006; Rushing & Selim 2019). These reactive species bind to the macromolecules causing toxicity and mutations, which lead to lipid peroxidation, necrosis, cell damage, cell death, deoxyribonucleic acid (DNA) lesions, carcinogenicity and other genetic diseases (Bedard & Massey 2006; Zarev et al. 2020).

Aflatoxin B_1_ deleteriously affects global public health as it is the major contributor to the worldwide occurrence of hepatocellular carcinoma (HCC). The toxin also works synergistically with hepatitis B and C viruses to significantly increase the risk of HCC far above either factor individually (Rushing & Selim 2019). Surgery and liver transplant offer limited treatment options as they are only useful in the treatment of early stages of HCC. Other non-surgical options such as chemo- and radio-therapies are only successful in patients with localised liver tumour (Darvesh, Aggarwal & Bishayee 2012). Therefore, research that focuses on the development of alternative preventative and therapeutic strategies may lead to valuable findings that may be effective in the control of HCC. Although the principal toxic target of AFB_1_ is the liver, several reports showed that exposure to AFB_1_ has adverse renal effects as it increases the levels of creatinine and urea in animal experiments (El-Mahalaway 2015; Yilmaz et al. 2018). Previous studies also revealed that oxidative stress resulting from AFB_1_ consumption leads to myocardial cell membrane destruction manifested by a marked increase in lactate dehydrogenase (LDH) activity (Mannaa et al. 2014).

The biosynthesis and activation of AFs can be attenuated using bioactive compounds present in herbal products. Plant extracts have been studied for their antifungal growth inhibition and mycotoxin detoxification. Several studies have been conducted on the protective effects of plant extracts and its bioactive compounds against AFB_1_-induced hepatotoxicity (Choi et al. 2010; Soni et al. 1997; Tang et al. 2007). Curcumin and ellagic acid are examples of phytochemicals isolated from plants and they inhibited hepatocarcinogenicity in rat and chicken models (Gowda et al. 2008; Soni et al. 1997). These compounds are known for their potent free radical scavenging ability and exert their anticarcinogenic effect primarily through prevention of oxidative stress (Darvesh et al. 2012). Furthermore, phytochemicals such as lycopene and quercetin have been reported to inhibit biotransformation of AFB_1_ toxicity by inducing detoxification enzymes (Choi et al. 2010; Tang et al. 2007).

*Monanthotaxis caffra* (Annonaceae) is a shrub occurring in evergreen forests of Eastern Cape, KwaZulu-Natal and Mpumalanga provinces of South Africa (National Research Council 2008). This plant species has been used in traditional medicine against tumours, microbial and parasitic infections in veterinary and human health (Mulholland et al. 2000; Okhale et al. 2016). Methanolic leaf extract of *M. caffra* has been also reported for its antigenotoxic properties against AFB_1_-induced genotoxicity (Makhuvele et al. 2018a). Furthermore, Crotepoxide, a known antitumour compound and other polyoxygenated cyclohexane derivative, 5,6-diacetoxy1-benzoyloxymethyl-1,3-cyclohexadiene were isolated from *M. caffra* (Makhuvele et al. 2018b; Mulholland et al. 2000). The *in vivo* hepatoprotective effects of *M. caffra* against mycotoxin-induced toxicity has never been explored. Therefore, this study aimed to investigate the protective effects of methanolic leaf extracts of *M. caffra* (MLEMC) against AFB_1_-induced hepatotoxicity on rats.

## Materials and methods

### Chemicals

Aflatoxin B_1_, dimethyl sulfoxide (DMSO) and curcumin were purchased from Sigma-Aldrich (St. Louis, United States). Methanol and acetonitrile were bought from Van Waters & Rogers Inc. (Radnor, USA) VWR and propylene glycol was from Merck (Darmstadt, Germany).

### Preparation of methanolic leaf extract of *M. caffra*

Leaves of *M. caffra* (Annonaceae) were collected from Lowveld National Botanical Gardens (South Africa) in March 2015. The identities of the plants were confirmed by Mrs. E. Van Wyk, University of Pretoria, South Africa. A voucher (number: PRU 122761 for *M. caffra*) was deposited in the H.G.W.J. Schweickerdt Herbarium of the University of Pretoria. The plant material was dried in an oven set at 45 °C and thereafter, ground to a fine powder. The powdered plant material was stored in airtight glass container in the dark at room temperature until use.

The powdered leaf material of *M. caffra* (350 g) was added to 3500 mL of 80% methanol and extracted to exhaustion by maceration at room temperature. The plant extracts were filtered through Whatman No. 1 filter paper and concentrated to dryness under reduced pressure using Buchi rotary evaporator.

### *In vivo* evaluation of hepatoprotective effect of methanolic leaf extracts of *M. caffra*

#### Animals

A total of 48 Sprague-Dawley male rats (7 weeks old), weighing between 150 g and 200 g, were obtained from South African Vaccine Producers (SAVP; Johannesburg, South Africa). The study was carried out using single gender as a way of reducing variation. During the experiment, the rats were kept in separate cages (2 per cage) under a controlled temperature of ±22 °C, and humidity at ±50% in a light and dark cycle of 12 h. The rats were fed with a conventional rodent diet and water, available *ad libitum* for the duration of the study. Rats were provided with enrichment including wooden sticks for gnawing, tissues and egg containers. All enrichment items and cages, water bottles and bedding were sterilised before use. Rats were acclimatised and closely monitored under laboratory conditions for five days prior to treatment.

#### Study design

The Sprague-Dawley rats were randomly divided into six groups of eight animals each (*n* = 8): Group A (healthy control, did not receive any treatment). Group B (negative control, received 25% propylene glycol), Group C (positive control, received curcumin dissolved in 25% propylene glycol (10 mg/kg body weight). The dose of curcumin was selected based upon previous studies where the dosage produced a marked hepatoprotective effects against aflatoxin B_1_ (Poapolathep et al. 2015). Group D, E and F rats were treated with 100 mg/kg, 200 mg/kg and 300 mg/kg body weight per day of MLEMC, respectively. The doses of the plant extracts were in line with those reported in literature (Sathya, Kokilavani & Ananta 2012). All treatments were administered once a day by oral gavage in the morning for 7 consecutive days. On day 8, all treated rats were administered 1 mg/kg b.w of AFB_1_ (Zarev et al. 2020), dissolved in reverse osmosis water by oral gavage, except the healthy control group. After three days, rats were sacrificed by using an isoflurane inhalation protocol for anaesthesia (Roustan, Perrin & Courbiere 2012).

#### Biochemical analysis

Blood samples were collected from the lateral tail vein from each rat on day 0 and by cardiac puncture on day 10 following anaesthesia with isoflurane prior to sacrifice for determination of serum biochemistry. The blood samples were centrifuged at 1500 × g, at 4 °C for 15 min and the serum was collected, then evaluated for serum enzyme level. Serum aspartate aminotransferase (AST), alanine aminotransferase (ALT), alkaline phosphatase (ALP), LDH and creatinine were measured using COBAS INTEGRA 400 kits from Roche following manufacturer’s instructions.

#### Histopathological studies

The liver was excised and then fixed in 10% buffered formalin for histological analysis. The organ was sliced and processed according to routine histology tissue processing in an automated tissue processor. After tissue processing, the sections were cut into 5 µm – 6 µm and the slides were prepared and stained in an automated haematoxylin and eosin tissue stainer before histology examination. The slides were examined with a BX 63 Olympus electron microscope with Olympus cellSens dimension version 1.12 software.

## Statistical methods

Data are presented as mean ± standard deviation. Differences between groups were determined using one-way analysis of variance (ANOVA). The standardised residuals were tested for deviations from normality using Dunnett’s, *t*-test and Shapiro–Wilk’s test. Least significant difference (LSD) test was used to determine statistical significance between the means of treated groups and the controls. The data were considered significant at *p* < 0.05.

## Results

### Effect of treatment on animal general conditions and body weight

Neither mortality nor clinical signs of illness and abnormalities were reported in all rats administered with MLEMC, negative (AFB_1_) and curcumin positive control treatments in this study. The administration of MLEMC did not affect the body weight, feed consumption and behavioural patterns of the rats at all tested concentration during 10 days of experiment. Based on these results, MLEMC can be considered safe for consumption by animals.

### Effect of treatment on serum biochemistry

**FIGURE 1:**
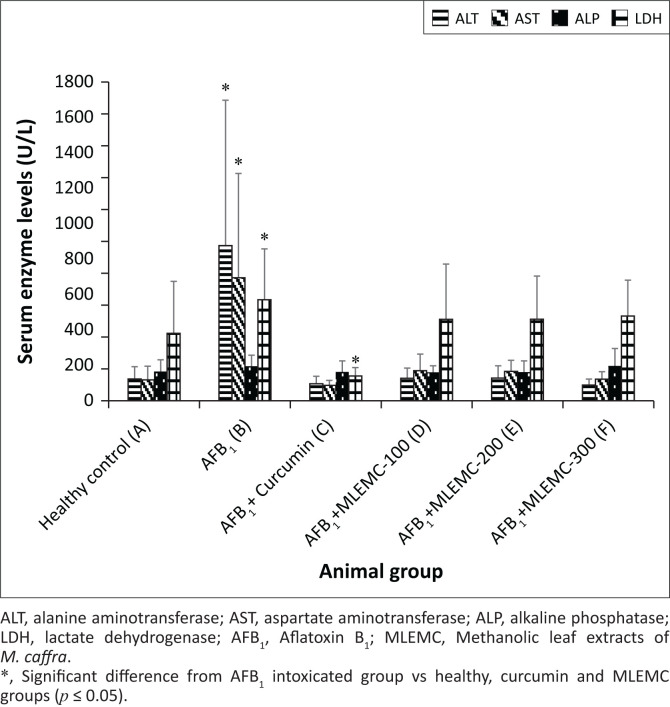
Effect of Methanolic leaf extracts of *M. caffra* on the serum levels of alanine aminotransferase, aspartate aminotransferase, alkaline phosphatase activities and lactate dehydrogenase following aflatoxin B_1_-induced hepatoxicity in rats.

**FIGURE 2:**
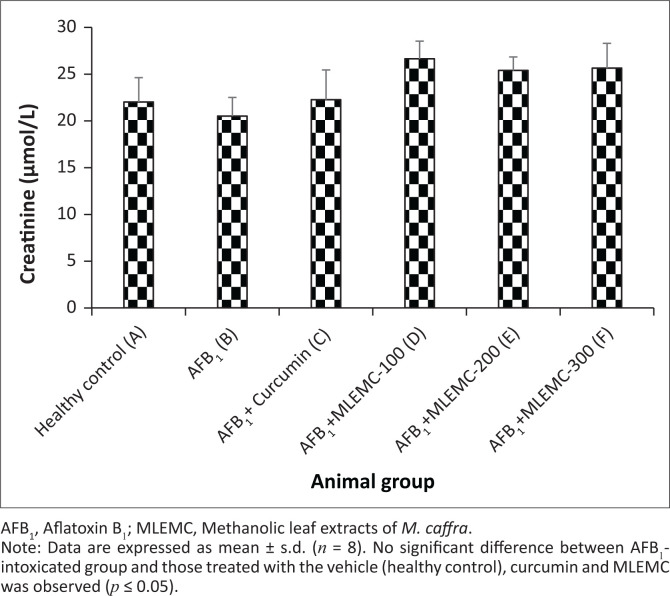
Effect of Methanolic leaf extracts of *M. caffra* on the levels of creatinine following aflatoxin B_1_-induced hepatotoxicity in rats.

Effects of different concentrations of MLEMC on serum levels in rats induced with AFB_1_ are presented in Figure 1. The levels of ALT and AST significantly increased in AFB_1_-treated rats (Group B) in comparison to the healthy and curcumin positive control groups (*p* < 0.05). The levels of these enzymes markedly decreased in rats administered with AFB_1_+MLEMC at different concentrations and the positive control curcumin (*p* < 0.05). Furthermore, there were no significant differences observed in ALT and AST levels between the healthy and curcumin control group and the AFB_1_+MLEMC treated groups. No significant differences were observed in the levels of ALP and creatinine between the healthy, AFB_1_ intoxicating group and curcumin control group and those treated with different concentrations of MLEMC (Figure 1). The LDH levels of AFB_1_-treated rats were significantly higher when compared to the LDH levels of healthy and curcumin treated group and the AFB_1_+MLEMC treated groups, which were not statistically different from each other (Figure 2). Moreover, a significant decrease in the LDH level in rats treated with curcumin was observed (*p* < 0.05).

### Effect of treatment on liver histopathology

**FIGURE 3:**
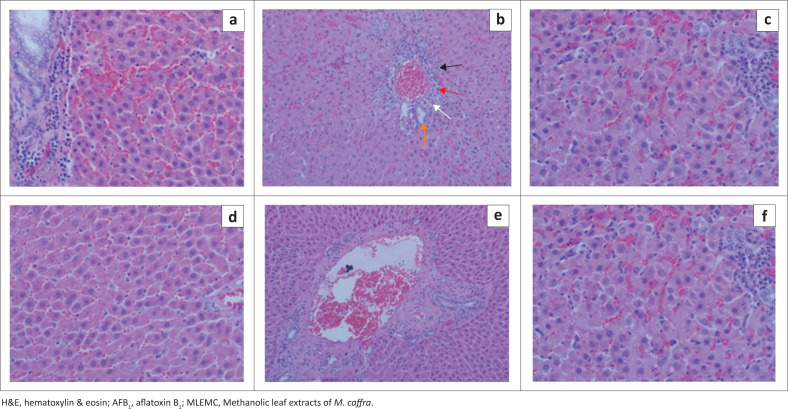
Effect of Methanolic leaf extracts of *M. caffra* on histology of the hepatocyte. The liver section was stained with H&E (40 ×). (a) Healthy control group; (b) Negative group; Showed mild to severe hepatocellular injury, the black arrow indicates bile duct proliferation, red arrow shows lymphoplasmacytic infiltrate, white arrow indicates fibrosis and orange arrow indicates normal bile duct; (c) AFB_1_+curcumin group showing normal Liver architect (d) AFB_1_+ MLEMC 100 mg/kg; (e) AFB_1_+MLEMC 200 mg/kg and (f) AFB_1_+MLEMC 300 mg/kg.

The effects of administration of MLEMC on liver histopathology are presented in Figure 3. Most rats in AFB_1_-intoxicated group (negative control) showed mild to severe hepatocellular degeneration with bile duct proliferation, hydropic degeneration, mild to moderate portal fibrosis in some of the larger tracts and portal lymphoplasmacytic infiltrates with the occasional neutrophil was observed in the periportal areas further extending into the periportal zone and disrupting the limiting plates, thus causing piecemeal necrosis. Scattered single cell hepatocellular necrosis was observed and the remaining hepatocytes had mildly granular and eosinophilic cytoplasm. The presence of phagocytic macrophages was also reported. These histological alterations were also observed in rats co-administered with AFB_1_+curcumin and AFB_1_+MLEMC but the injuries were substantially reduced to slight signs of sublethal non-specific hepatocellular injuries. Slight granular eosinophilic cytoplasms were also observed in all of the hepatocytes. Inflammation or necrosis were not observed and the portal tracts were within normal limits.

## Discussion

Human and animals are exposed to different xenobiotic substance such as aflatoxins that cause deleterious effects on the biomolecules and cellular membrane by inducing oxidative damage (El-Agamy 2010). The liver is an essential organ of human and animals, which is involved in metabolism of xenobiotics (Colakoglu & Donmez 2012). Aflatoxin B_1_ is a well-known hepatotoxic agent. It induces cellular and tissue damage to the hepatocytes, thus resulting in aflatoxicosis, HCC, necrosis, cirrhosis, etc. (Shan 2020; Zarev et al. 2020). Aflatoxin B_1_ mediates these various deleterious effects through the induction of oxidative stress (El-Agamy 2010). Methanolic leaf extracts of *M. caffra* ameliorated AFB_1_-induced genotoxicity in *in vitro* antigenotoxic assays (Makhuvele et al. 2018b). Due to this antigenotoxic effect of MLEMC, we hypothesised that MLEMC will ameliorate AFB_1_-induced hepatotoxicity in rats. Therefore, we further investigated the protective effects of MLEMC against AFB_1_-induced hepatotoxicity in rats.

In our study, AFB_1_ intoxication significantly increased the serum levels of the liver enzymes AST and ALT in comparison with untreated control group, which implies that AFB_1_ caused cellular damage to the liver because of the release of these enzymes, which are normally located in the cytoplasm of the liver cells, in the blood stream. The higher standard deviation observed in the AFB_1_ intoxicated group were because of the two rats which did not respond to AFB_1_, their serum levels were not afffected by administration of AFB_1_. In general, AST and ALT are released into the blood stream when there is a liver damage or loss of cell membrane intergrity. These serum enzymes are considered as common biomarkers for the diagnosis of liver damage (Amacher 1998). Alanine aminotransferase and AST were normal in healthy control group whilst co-adminsteration of AFB_1_+curcumin and AFB_1_+MLEMC (100 mg/kg, 200 mg/kg and 300 mg/kg) normalised the serum enzymes ALT and AST. No significant difference was observed between the MLEMC and curcumin-treated groups in comparison with the healthy control group. This is a clear indication that MLEMC and curcumin ameliorated the toxic effect of the AFB_1_, by inhibiting AFB_1_-induced liver cell injury damage and consequently lowering the AST and ALT levels in the blood. Curcumin was used as a positive control in this study as it is renowned for its protective effects against AFB_1_-induced hepatocarcinogenesis and hepatotoxicity in rodents, broilers and ducklings by lowering the levels of serum markers and lipid peroxidation (Chuang et al. 2000; Gowda et al. 2008; Mathuria & Verma 2008). Methanolic leaf extracts of *M. caffra* demonstrated protective effect against AFB_1_-induced toxicity at all tested concentrations. However, the highest concentration of MLEMC showed high reduction comparably with the lower concentration, although no significant difference was observed.

An increase in LDH levels in rats intoxicated with AFB_1_ only was observed as compared with untreated control. Increased LDH activity is one of the major events involved in liver damage after AFB_1_ administration, as it is only released in the blood stream when the liver is injured (Choi et al. 2010; Nayak & Sashidhar 2010). However, this increase in LDH activity was not observed in the healthy control and in rats treated with different concentrations of AFB_1_+MLEMC. A significant decrease in the LDH level in rats treated with curcumin was observed, which is in line with literature reports (Nayak & Sashidhar 2010).

Creatinine, a major marker of kidney function, is the final metabolite of creatine conversion. Increased creatinine as an index of impaired kidney function because of chronic exposure to AFB_1_ was reported in chickens and rats (El-Mahalaway 2015; Valchev et al. 2014). In this study, significant changes were not observed in the serum level of creatinine in rats treated with a single dose of AFB_1_, which is in line with previous reports where nephrotoxicity was observed only in animals having chronic exposure to AFB_1_ (El-Mahalaway 2015; Valchev et al. 2014).

The result of biochemical analysis of the protective effects of MLEMC was also confirmed by the histopathological investigations. Aflatoxicosis is characterised by hydropic changes, vacuolar degeneration, bile duct proliferation and lymphoplasmacytic infiltration in exposed hepatocytes. However, the histopathological effects of AFB_1_ are directly proportional to the concentration and exposure time to AFB_1_ (Do & Choi 2007; Yaman, Yener & Celik 2016). Aflatoxin B_1_ intoxication caused mild to severe hepatocellular injury accompanied by bile duct proliferation, hydropic changes and lymphoplasmacytic infiltrate in the hepatocytes of exposed rats. This histological effect of AFB_1_ on rat hepatocytes has been reported in literature (Yaman et al. 2016). Minimal degree of the above-mentioned histological characteristics in rats exposed to co-administered AFB_1_+curcumin and AFB_1_+MLEMC were observed in this study, thus implying that curcumin and MLEMC had recuperative effects against AFB_1_-induced acute toxicity. Curcumin has been reported in literature to possess ameliorative effects on the histology of the liver and other organs (El-Agamy 2010; Gowda et al. 2008).

Members of the genus *Monanthotaxis* are known to contain essential oils (Parmena, Mgina & Joseph 2012), alkaloids, flavonoids and cyclohexane epoxides (Mulholland et al. 2000). Chemical analysis of the 80% extract of *M. caffra* revealed it is rich in cyclohexane epoxide derivatives including crotepoxide and 5,6-diacetoxy1-benzoyloxymethyl-1,3-cyclohexadiene and a mixture of two related benzoyloxy cyclohexidiene derivatives (Makhuvele et al. 2018b). Bioassay-guided fractionation of the extract using VITOTOX genotoxicity assay yielded the two antimutagenic compounds crotepoxide and 5,6-diacetoxy1-benzoyloxymethyl-1,3-cyclohexadiene. In addition to their antimutagenic effects against aflatoxin B_1_-induced mutagenicity in Ames and Vitotox assays, the antitumour or anticarcinogenic properties of these polyoxygenated cyclohexane derivatives have been reported in literature. Crotepoxide inhibited the expression of tumour necrosis factor (TNF) regulated gene products involved in anti-apoptosis such as Bcl-2, Bcl-XL, cyclin D1, Cox-2, Bax, Bid, c-Myc, MMP-9 and VEFG, etc. Furthermore, crotepoxide also inhibited the tumours by preventing the activation of genes that are involved in tumorigenesis at gene levels (Alonso-Amelot 2016). The compound also possessed antitumour properties against various carcinoma in rats and mice (Parmena et al. 2012; Shing & Tam 1996; Starks et al. 2012). The protective effects of these plant extracts against AFB_1_ hepatotoxicity may be due to the presence of the above-mentioned compounds, which are found in Annonaceae plant species.

## Conclusion

The present study showed that pre-treatment of rats with MLEMC protected hepatocytes from AFB_1_-induced hepatotoxicity as evidenced by the normalisation of the serum enzyme levels of AST, ALT and LDH and the reduction of hepatocellular lesions. This effect was comparable to that produced by curcumin a known hepatoprotective agent against AFB_1_-induced toxicity. This hepatoprotective effects results from the synergistic effects of the compounds present in the MLEMC. Our results suggest that MLEMC can be considered as potential natural agent for prevention of AFB_1_-induced hepatotoxicity. Further studies on long time treatment of MLEMC against AFB_1_-induced hepatotoxicity in rats are recommended.
